# S-glutathionylation modification of proteins and the association with cellular death (Review)

**DOI:** 10.3892/mi.2025.263

**Published:** 2025-08-22

**Authors:** Xiongxing Sun, Le Xie, Shiliang Wang, Shanshan Zeng, Lingying Wu, Xukun Tang, Jiajian Zhu, Shigao Lin, Tenghui Hu, Lin Jia, Xia Li, Songqing Zhang, Jun Deng, Dahua Wu

**Affiliations:** 1 Graduate School of Hunan University of Chinese Medicine, Changsha, Hunan 410208, P.R. China; 2 Hunan Provincial Hospital of Integrated Traditional Chinese and Western Medicine (The Affiliated Hospital of Hunan Academy of Traditional Chinese Medicine), Changsha, Hunan 410060, P.R. China; 3 Shimen County Traditional Chinese Medicine Hospital, Changde, Hunan 415300, P.R. China; 4 Xiangxi Tujia and Miao Autonomous Prefecture Ethnic Traditional Chinese Medicine Hospital, Jishou, Hunan 416000, P.R. China

**Keywords:** S-glutathionylation, redox signaling, glutaredoxins, programmed cell death, oxidative stress

## Abstract

S-glutathionylation (SSG), a redox-sensitive post-translational modification mediated by glutathione, regulates protein structure and function through reversible disulfide bond formation at cysteine residues. Glutaredoxins (GRXs), pivotal antioxidant enzymes, catalyze SSG dynamics to maintain thiol homeostasis. Recent advances in redox proteomics have revealed that SSG dysregulation is intricately linked to neurodegenerative, cardiovascular, pulmonary and malignant diseases. Notably, GRX isoforms (GRX1 and GRX2) play compartment-specific roles in disease pathogenesis: GRX1 modulates hepatic lipid metabolism and pulmonary fibrosis, while GRX2 sustains mitochondrial redox balance and Fe-S cluster assembly. Notably, SSG functions as a ‘double-edged sword’ in programmed cell death (PCD). While moderate SSG protects against irreversible cysteine oxidation, persistent SSG accumulation due to GRX dysfunction triggers apoptosis, necroptosis and ferroptosis by disrupting redox-sensitive targets, such as caspases, BAX and glutathione peroxidase 4. The present review summarizes, for the first time, at least to the best of our knowledge, the association of SSG with distinct PCD subtypes, and highlights therapeutic strategies targeting GRX activity or site-specific SSG modulation (e.g., pyruvate kinase M2 Cys423/424). Emerging approaches, including GRX mimetics and thiol-targeted drugs, hold promise for precision medicine in redox-related pathologies.

## 1. Analysis of protein S-glutathionylation

Biological oxidation, the process of energy substrate breakdown, generates ATP and reactive oxygen species (ROS), including superoxide anion (O_2_^-^), hydroxyl radical (·OH) and hydrogen peroxide (H_2_O_2_). While low ROS levels sustain redox equilibrium, excessive ROS induce oxidative stress, disrupting lipid membranes, proteins, DNA and cytoskeletal integrity (1). Mitochondria-derived ROS are pivotal in redox signaling and pathology (2). Cellular redox balance relies on enzymatic [e.g., superoxide dismutase and glutathione peroxidase (GPX)] and non-enzymatic [e.g., glutathione (GSH)] antioxidants to neutralize ROS (3). The dysregulation of oxidant-antioxidant systems underpins diverse diseases, from neurodegeneration to cancer.

### Structure and biological roles of GSH

GSH, a γ-glutamyl-cysteinyl-glycine tripeptide, is the primary endogenous antioxidant. Synthesized via glutamate-cysteine ligase (the rate-limiting enzyme) and GSH synthetase, GSH functions as a key antioxidant. It scavenges ROS through the nucleophilic attack by the thiol group (-SH) of its cysteine residue (4). ROS attack protein thiols, forming sulfenic acid (-SOH), which may progress to irreversible sulfinic (-SO_2_H) or sulfonic (-SO_3_H) acids, impairing protein function (5). GSH combats oxidative stress by directly scavenging ROS (e.g., H_2_O_2_, ONOO^-^) and by reducing oxidized thiol groups on other molecules (6,7).

The GSH/GSH disulfide (GSSG) ratio reflects cellular redox status: a high ratio (>100:1) signifies a reduced state, while under severe oxidative stress, this ratio is markedly reduced to a range of 1:1 to 10:1(8). Tissue-specific GSH levels vary, peaking in the liver (highest detoxification demand) and declining in adipose tissue (9). Compartmental redox potentials further regulate function; for example, the endoplasmic reticulum maintains an oxidizing redox environment (approximately -180 mV), characterized by a lower GSH/GSSG ratio (typically 1:1-3:1) compared to the cytosol (approximately -220 to -260 mV; GSH/GSSG >100:1), facilitating disulfide bond formation during protein folding (10).

### Protein oxidative thiol modifications. Types of thiol oxidative modifications

**Figure 1:**
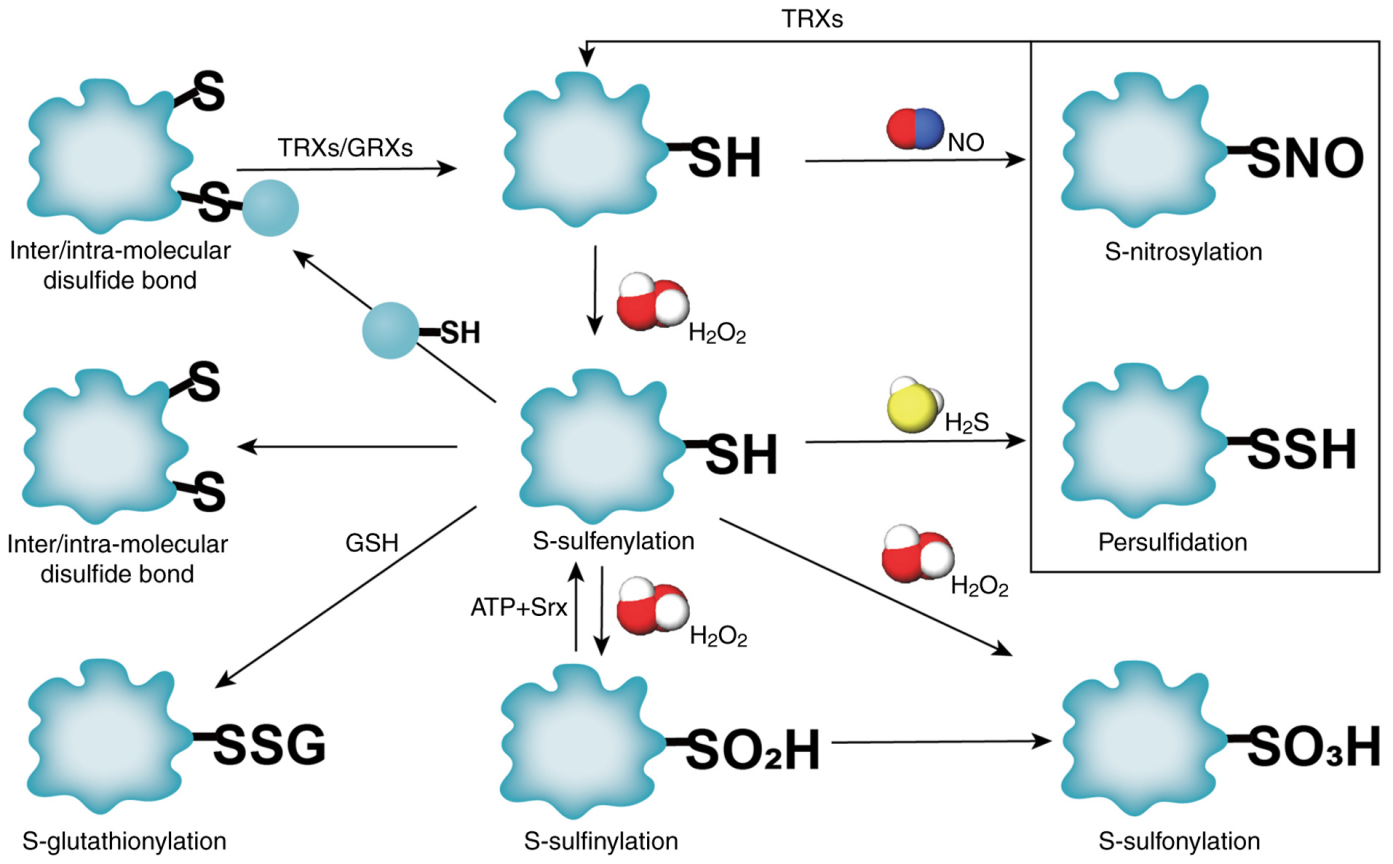
Regulation of different OxiPTM thiol reaction steps and the cysteine ‘oxidation-reduction switch’ control system. OxiPTM, oxidative post-translational modification; TRX, thioredoxin; GRX, glutaredoxin; SSG, S-glutathionylation; -SSH, persulfides; SNO, S-nitrosylation.

ROS induce dynamic oxidative post-translational modifications on cysteine residues, serving as redox switches to regulate protein conformation, signaling, and cellular homeostasis (11). Key reversible modifications include the following: i) S-glutathionylation (SSG), where GSH adduct formation occurs via thiol-disulfide exchange. ii) S-nitrosylation (SNO), where nitric oxide (NO)-mediated covalent binding to thiols occurs, modulating synaptic transmission and mitochondrial function (12). iii) S-sulfenylation (SOH), where the initial oxidation product of cysteine by H_2_O_2_, functions as a precursor for SSG or disulfide bonds (13). These modifications are tightly regulated by reductases [e.g., S-nitrosoglutathione (GSNO) reductase for SNO removal] and cellular redox state (14). Persistent oxidative stress drives irreversible oxidation to -SO_2_H or sulfonic-SO_3_H acids, disrupting protein function and promoting pathological outcomes (e.g., neurodegeneration and metabolic dysfunction) (15). Gasotransmitters, such as NO and H_2_S, further fine-tune redox signaling: H_2_S preferentially reacts with SOH to form persulfides (-SSH), protecting thiols from overoxidation (13). Key metabolic enzymes regulate these processes, as depicted in Fig. 1.

**Figure 2:**
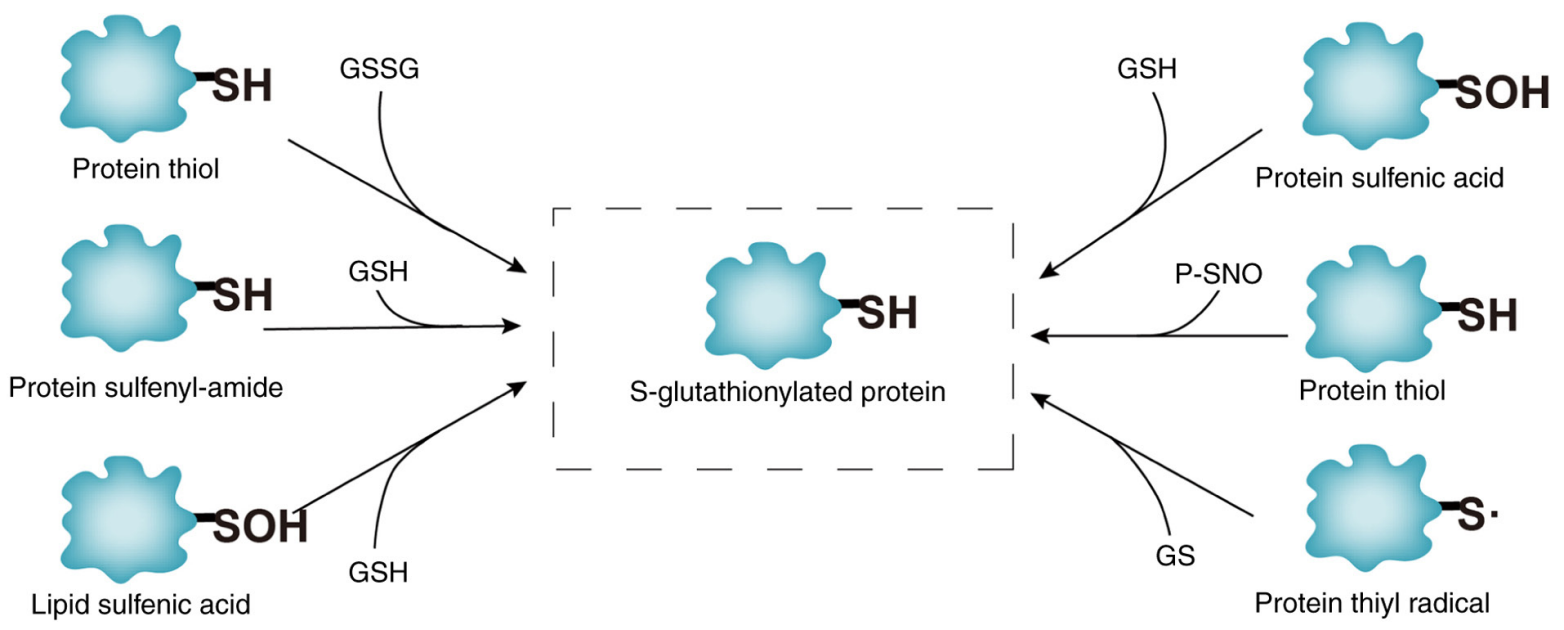
Different biochemical mechanisms of protein SSG modification. SSG, S-glutathionylation; -SOH, sulfenic acid; GSH, glutathione; GSSG, GSH/GSH disulfide.

*Molecular mechanisms of protein SSG modification*. SSG modification, mediated by GSH, involves the covalent attachment of GSH to cysteine thiols, forming mixed disulfide bonds. This redox-sensitive process regulates protein activity, protects cysteine residues from irreversible oxidation and maintains thiol/disulfide balance (16). As illustrated in Fig. 2, SSG formation occurs through six pathways: i) Thiol-disulfide exchange: Direct reaction between protein thiols and oxidized glutathione (GSSG). ii) SOH intermediates: Protein sulfenic acid (P-SOH) reacts with GSH or GSNO (GSH-SOH). iii) Sulfinamide intermediates: Thiol metabolites (e.g., P-SNOR) interact with GSH. iv) Sulfur radical pathways: ROS/RNS generate sulfur radicals (S·), leading to disulfide fomation. v) SOH lipid mediation: Lipid-SOH intermediates react with GSH. vi) SNO intermediates: GSNO facilitates the conversion of thiol-nitrosylated proteins (P-SNO) to SSG (17). Although intermediates vary across pathways, all converge on redox exchange between GSH/GSSG and protein thiols, as depicted in Fig. 2. SSG dynamics are critical for redox signaling and stress adaptation, with glutaredoxins (GRXs) playing a central role in reversing these modifications (as described below).

### GRXs in the regulation of SSG modifications

GRXs, first identified in *Escherichia coli* by Holmgren in 1976(18), are GSH-dependent oxidoreductases critical for reversing SSG (19). GRXs catalyze deglutathionylation via two mechanisms: The monothiol pathway (single active-site cysteine reacts with GSH to release GSSG) and the dithiol pathway (two cysteines form intermolecular disulfides) (20). Their conserved Cys-X-X-Cys motif enables binding to GSH; however, it limits the reduction of sulfonic acids (-SO_3_H) or intermolecular disulfides (21).

Mammals express two isoforms: GRX1 (cytoplasmic) and GRX2 (mitochondrial/nuclear), sharing 34% homology (22). GRX1 predominantly utilizes the monothiol pathway, maintaining iron homeostasis and 2Fe-2S cluster assembly, essential for electron transport and anti-apoptotic functions (23). GRX2, although less abundant, exhibits enhanced Fe-S cluster synthesis and mitochondrial SSG regulation, sustaining antioxidant capacity under oxidative overload (24). Both isoforms dynamically regulate SSG levels based on cellular GSH/GSSG ratios. Oxidative stress reduces this ratio, overwhelming GRX activity and driving irreversible protein oxidation linked to cell death (25).

Despite the non-essential role of GRX1 in viability (GRX1^-^/^-^ mice exhibit a normal lifespan under standard conditions) (26), as it modulates disease-specific pathways. For example, GRX1 deficiency exacerbates hepatic lipid dysregulation via sirtuin1 glutathionylation (27), while GRX2 loss impairs mitochondrial redox balance, accelerating lens epithelial-mesenchymal transition and cataract formation through ILK/AKT/GSK-3β dysregulation (28). Therapeutic restoration of GRX1 in pulmonary fibrosis models reduces pathological SSG accumulation, highlighting its potential as a redox-targeted therapy (24).

## 2. Thiol redox modulations in programmed cell death

Programmed cell death (PCD), including apoptosis, necroptosis, pyroptosis, autophagy and ferroptosis, is a genetically regulated process essential for tissue homeostasis and pathogen defense (29). In contrast to accidental necrosis, PCD eliminates superfluous or damaged cells through precise molecular cascades (e.g., caspase activation, inflammasome signaling). Cellular redox imbalance, driven by excessive ROS, disrupts thiol homeostasis by oxidizing critical cysteine residues in proteins (e.g., caspases, BAX and GPX4) and small molecules such as GSH (30). Reversible thiol modifications, particularly SSG, act as redox switches to regulate PCD execution. For instance, SSG modulates death receptor signaling (e.g., FAS activation), caspase activity, and antioxidant defense systems (e.g., GPX4 in ferroptosis), as summarized in Table I (31).

SSG dynamically balances pro-survival and pro-death signals. Under oxidative stress, diminished GRX activity impairs deglutathionylation, leading to persistent SSG accumulation. This disrupts redox-sensitive pathways (e.g., RAS/ERK and NLRP3 inflammasome) and shifts cellular fate toward PCD (32). GRX dysfunction further exacerbates mitochondrial SSG overload, impairing electron transport chain complexes and amplifying ROS-driven damage (33). Thus, SSG serves as both a protective mechanism (shielding thiols from irreversible oxidation) and a pathogenic trigger (sustaining oxidative stress), depending on cellular context and modification dynamics.

### SSG modification and cell apoptosis

Apoptosis, characterized by cell shrinkage, chromatin condensation and caspase activation, is regulated through intrinsic (mitochondrial), extrinsic (death receptor) and endoplasmic reticulum (ER) stress pathways (34). The intrinsic pathway involves mitochondrial permeability transition pore (MPTP) opening, controlled by BCL-2 family proteins (e.g., BAX/BAK). Oxidative stress induces SSG of BAX at Cys62, promoting mitochondrial translocation and caspase-9/3 activation, although the conformational effects remain unclear (35). GRX1 overexpression mitigates apoptosis in myocardial infarction models by restoring BCL-2/BAX balance (36).

The extrinsic pathway is initiated by death receptors (e.g., FAS and TNFR) with redox-sensitive cysteine-rich extracellular domains. SSG of FAS at Cys294 enhances FASL binding, accelerating caspase-8/3 activation (37). Similarly, SSG of pro-caspase-3 (Cys184/220) inhibits its activation, while TNF-α-induced GRX1 downregulation shifts this balance toward apoptosis (38). In ethanol-exposed GRX1-deficient mice, FAS-SSG accumulation drives hepatocyte apoptosis via NF-κB and AKT dysregulation (39).

ER stress-mediated apoptosis arises from misfolded protein aggregation and Ca²^+^ imbalance, activating CHOP and JNK pathways (40). SSG of ER chaperones (e.g., BiP) at Cys420/441 modulates ATPase activity and protein folding, paradoxically suppressing myeloma cell apoptosis (41). Conversely, glutathione S-transferase Pi (GSTP)1 promotes ER stress-induced apoptosis in liver cancer via the glutathionylation of calreticulin and sarco/endoplasmic reticulum Ca^2+^-ATPase (SERCA), inhibiting JNK survival signals (42). While SSG generally enhances apoptosis, its role in ER protein quality control may contextually oppose cell death.

### SSG modification and autophagy

Autophagy, a lysosome-dependent degradation process, includes macroautophagy, microautophagy and chaperone-mediated autophagy, playing dual roles in cell survival and death (43). Oxidative stress (e.g., hypoxia and ischemia/reperfusion) induces autophagy to degrade damaged organelles and proteins, while autophagy itself regulates redox balance by clearing oxidized products (44). SSG functions as a redox switch in autophagy modulation. For instance, H_2_S-induced SSG of KEAP1 at Cys434 disrupts KEAP1-NRF2 binding, promoting autophagy gene expression (45). Conversely, GSH depletion in cancer cells reduces SSG, leading to oxidative stress and autophagy activation (46). The loss of KRIT1 increases the SSG of chaperones and cytoskeletal proteins, impairing autophagic flux and quality control (47). Oncogenic H-RAS12 enhances GAPDH SSG, depleting GSH and triggering autophagy-independent of ROS accumulation (48).

SSG also negatively regulates autophagy. The SSG of ATG3 and ATG7 inhibits LC3 lipidation, a critical step in autophagosome maturation (49). Similarly, the SSG of AMPKα (Cys299/304) and SENP3 (Cys243/274) disrupts Beclin1 and PtdIns3K complex formation, impairing autophagosome assembly (50). PTEN SSG at Cys124/71 inhibits its phosphorylation, activating AKT/mTOR and suppressing autophagy, potentially driving pathological cell proliferation (51). Conversely, the SSG of ATM at Cys2991 promotes peroxisomal autophagy via PEX5 ubiquitination (52). The MiT/TFE transcription factors (e.g., TFEB) are regulated by SSG, enhancing lysosomal biogenesis and autophagy initiation (53).

GRXs are central to autophagy regulation by reversing SSG. GRX1 deficiency in liver cancer cells increases oxidative modifications and autophagic flux, while PRDX6-mediated GRX1 upregulation suppresses autophagy (54). GRX1 also protects against oxidative stress by maintaining AKT phosphorylation and mTORC1 activity, inhibiting autophagy in ischemic myocardial cells (55). GRX2, crucial for mitochondrial autophagy, stabilizes mitochondrial dynamics and ultrastructure. GRX2^-^/^-^ mice exhibit reduced GSH/GSSG ratios and increased mitophagy, highlighting its role in redox homeostasis (33). These findings underscore the ‘thiol switch-autophagy cascade’ as a therapeutic target in diseases like cancer and neurodegeneration (56).

### SSG modification and other forms of cell death

Necroptosis, a caspase-independent PCD, is triggered by RIPK3-mediated phosphorylation of MLKL, leading to membrane rupture and inflammatory damage-associated molecular pattern release (57). The SSG of mitochondrial fusion protein MFN2 disrupts mitochondria-ER crosstalk, promoting necroptosis in cadmium-induced neurotoxicity. This modification is reversible by cytoplasmic GRX1, but not mitochondrial GRX2, highlighting compartment-specific redox regulation (58). Caspase-8, a necroptosis inhibitor, undergoes intermolecular SSG at Cys360/409 under low thiocyanate conditions, impairing its ability to suppress RIPK3 phosphorylation (59). In models of Parkinson's disease (PD), GRXs paradoxically enhance microglial necroptosis via TNF-α/NF-κB upregulation, suggesting context-dependent roles (60).

Pyroptosis, driven by gasdermin D (GSDMD) cleavage and pore formation, amplifies inflammation through the release of IL-1β (61). Redox modifications regulate pyroptosis: The SSG of NLRP3 at Cys483 inhibits inflammasome activation, while thioredoxin (TRX)-1 reduces NLRP3 cysteine reactivity, attenuating sepsis-induced pyroptosis (61). The active thiols of GSDMD (Cys38/56/268/467) are susceptible to oxidative modifications that enhance caspase-1-mediated cleavage, linking mitochondrial ROS to inflammatory death (63). However, the mechanistic interplay between glutathionylation and pyroptosis remains underexplored.

Ferroptosis, an iron-dependent lipid peroxidation process, is tightly linked to GSH metabolism. System Xc^-^ (SLC7A11-dependent cystine uptake) sustains GSH synthesis, while GPX4 utilizes GSH to neutralize lipid hydroperoxides (64). GRX2, via Fe-S cluster assembly, mitigates ferroptosis by maintaining mitochondrial redox balance (65). GSH depletion (e.g., erastin treatment) or GRX5 silencing induces iron overload and ferroptosis, sensitizing cancer cells to chemotherapy (66). In hereditary ataxia, FXN deficiency impairs Fe-S biogenesis, reducing GRX/TRX activity; NRF2 activators (e.g., sulforaphane) may counteract this defect (67).

A newly identified death modality, disulfidptosis, arises from NADPH depletion-induced disulfide stress. SLC7A11 overexpression under glucose starvation promotes aberrant actin cytoskeleton SSG, triggering cytoskeletal collapse and membrane detachment (68). Unlike ferroptosis or apoptosis, disulfidptosis is uniquely potentiated by thiol oxidants (e.g., diamide) and unresolved by GRX-mediated redox repair, suggesting distinct therapeutic vulnerabilities.

## 3. Protein SSG modifications in disease pathogenesis

SSG regulates cell growth, differentiation and apoptosis by modulating enzymatic activity, protein conformation and stability through redox-sensitive mechanisms (69). Advances in redox proteomics have linked SSG dysregulation to neurodegenerative, cardiovascular, respiratory and malignant diseases, as summarized in Table II.

### Neurodegenerative diseases

Mitochondrial oxidative stress in Alzheimer's disease (AD) drives Aβ accumulation and tau hyperphosphorylation, exacerbating neuronal damage (70). Reduced GSH/GSSG ratios in patients with AD are associated with disease severity, while SSG levels of cortical proteins (e.g., GAPDH and α-enolase) are elevated, impairing synaptic function (71). The expression of GRX1 and GRX2 is markedly reduced in the brains of patients with AD, particularly in the CA1 region, contributing to F-actin destabilization and memory deficits (72). APP/PS1 transgenic mice overexpressing GRX1 exhibit a restored synaptic plasticity and cognitive function, highlighting therapeutic potential (73).

PD is characterized by dopaminergic neuron loss and α-synuclein aggregation, exacerbated by GSH depletion in the substantia nigra (74). There is evidence to indicate that oxidative stress induces the SSG of α-synuclein, which alters its conformational stability and promotes pathological oligomerization (75). GRX1 deficiency exacerbates this process, leading to enhanced α-synuclein toxicity and dopaminergic neurodegeneration in *C. elegans* models of PD (76). The SSG of DJ-1 at Cys106 enhances its mitochondrial localization and ROS scavenging, whereas GRX1 upregulation in MPTP models paradoxically accelerates respiratory chain dysfunction and neuron death (77). Conversely, GRX2 promotes Fe-S cluster assembly, mitigating oxidative damage, while Parkin deglutathionylation rescues proteasomal dysfunction, underscoring context-dependent GRX roles (78).

### Cardiovascular system diseases

In cardiomyocytes, mitochondrial and sarcoplasmic reticulum redox dynamics are critical for energy metabolism and contractility. GRX2 mitigates oxidative stress by reducing SSG of NADPH oxidase subunits (NDUFS1/NDUFV1), suppressing mitochondrial ROS overproduction linked to left ventricular hypertrophy and hypertension (79). The sarcoplasmic reticulum Ca²^+^ channel RyR2 undergoes SSG under oxidative stress, which initially protects against calcium overload in ischemia-reperfusion injury, whereas it becomes maladaptive in sustained oxidative environments (e.g., catecholamine-induced arrhythmias) (80). The R2474S RyR2 mutation exacerbates SSG-mediated mitochondrial oxidation, increasing ventricular arrhythmia susceptibility (81).

Atherosclerosis progression is associated with low-density lipoprotein ApoB100 SSG levels, promoting endothelial dysfunction and plaque instability (82). GRX1 inhibition (e.g., 2-AAPA) attenuates endothelial-mesenchymal transition by reducing SSG, suggesting therapeutic potential in vascular remodeling (83). In myocardial infarction, the SSG of SERCA and Na^+^/K^+^ ATPase α-subunit impairs calcium handling and action potential generation, exacerbating contractile dysfunction and oxidative damage (46). Pharmacological agents such as ergothioneine acid reduce the SSG of NF-κB-dependent Wnt5a-sFlt1, improving post-MI outcomes by preserving GRX1 activity and myocardial integrity (84).

### Respiratory system diseases

Chronic obstructive pulmonary disease (COPD), driven by exogenous oxidants (e.g., cigarette smoke) and endogenous ROS/nitric oxide synthase (NOS), is characterized by macrophage/neutrophil infiltration, epithelial cell death and fibrosis (85). Cigarette smoke-induced SSG accumulation in lung proteins promotes alveolar epithelial apoptosis, while GRX1 overexpression rescues cell survival and reduces airway inflammation (86). GRX1 deficiency exacerbates COPD progression by elevating TGF-β levels, collagen deposition and basal cell plasticity, accelerating lung remodeling (87).

In acute lung injury (ALI), oxidative stress reduces GRX1 expression, impairing redox homeostasis. The SSG of FABP5 at Cys127 activates PPARβ/δ, suppressing macrophage inflammation and alleviating H_2_O_2_-induced ALI (88). Asthma pathogenesis involves NF-κB-mediated airway inflammation, where the SSG of IKKβ at Cys179 inhibits pro-inflammatory chemokine production (89). GRX1^-^/^-^ mice exhibit attenuated LPS-induced cytokine release (IL-1β and TNF-α) and macrophage dysfunction, suggesting the dual role of GRX1 in the regulation of inflammation (90). Notably, the SSG of IL-1β at Cys188 directly reduces its inflammatory activity, highlighting a self-limiting redox checkpoint (91). The therapeutic administration of recombinant GRX1 reverses pathological SSG, providing promise for chronic lung diseases (92).

### Malignant tumors

Cancer cells exhibit metabolic reprogramming (Warburg effect) and redox adaptation to sustain proliferation under oxidative stress. SSG modulates tumor progression by regulating enzymes critical for glycolysis, drug resistance and protein stability (93). For example, SSG of pyruvate kinase M2 (PKM2) at Cys358 suppresses its activity, attenuating glycolytic flux and oxidative stress in small-cell lung cancer (94). Conversely, the SSG of transglutaminase 2 at Cys193 promotes its degradation, sensitizing colon cancer cells to 5-fluorouracil by restoring apoptosis (95).

Clinically, SSG levels of plasma proteins (e.g., serpin A1/A3) are associated with radiotherapy efficacy in prostate cancer, serving as potential biomarkers (96). In breast cancer, oxidized Hsp90 (with reduced SSG) is associated with a poor treatment response, while the SSG of GSTP at Cys411/420 counteracts bortezomib resistance in multiple myeloma by impairing ATPase binding and protein folding (97). These findings position SSG as a dual regulator of tumor survival and therapeutic vulnerability, with targeted cysteine modification offering novel avenues for anticancer drug development.

The therapeutic targeting of SSG modifications confronts significant pharmacological constraints. Target specificity is hindered by functional divergence among GRX isoforms, particularly GRX1 which exhibits opposing effects in pulmonary protection vs. neurodegenerative exacerbation. The reversible instability of SSG modifications, demonstrated in the context-dependent roles of RyR2 glutathionylation during cardiac injury, compromises sustained efficacy. Direct clinical biomarkers for pathological SSG sites remain undeveloped, restricting clinical monitoring beyond indirect redox indicators such as GSH/GSSG ratios. Additionally, detection sensitivity suffers from technical limitations in capturing low-abundance labile SSG modifications *in situ*. Addressing these barriers will enhance the translational rigor of SSG-targeted therapies and strategically guide future mechanistic and therapeutic development.

## 4. Summary and future perspectives

ROS-mediated cysteine modifications, particularly SSG, serve as dynamic redox switches regulating cellular signaling, death pathways and stress adaptation. SSG dynamics are governed by the GSH/GSSG ratio, GRX activity and oxidative stress intensity. While moderate SSG protects against irreversible cysteine oxidation (e.g., -SOH/-SO_2_H), severe oxidative stress drives pathological overoxidation, triggering cell senescence or death (98). The complexity of thiol redox regulation necessitates advanced redox proteomics to map tissue- and site-specific SSG modifications, given their low abundance and instability (99).

SSG critically modulates diverse cell death modalities (apoptosis, autophagy, pyroptosis, etc.) by altering protein conformation, membrane integrity and mitochondrial function, often amplifying inflammatory cascades (31). Therapeutic strategies targeting SSG show promise: Recombinant TRX or N-acetylcysteine reduces ischemic reperfusion injury by restoring endothelial NOS activity (100), while tanshinone IIA, a traditional Chinese medicine component, protects against myocardial ischemia via PKM2 glutathionylation at Cys423/424(101). Glutaredoxin mimetics, such as para-aminobenzoic acid-conjugated glutaredoxin peptide enhance deglutathionylation activity, showing efficacy in reducing pathological SSG accumulation in pulmonary fibrosis models by emulating the catalytic function of GRX1(24). Complementary thioredoxin mimetics exemplified by TXM-CB3 indirectly support GRX systems through the replenishment of reducing equivalents, attenuating mitochondrial SSG overload in neurodegenerative contexts (78). Concurrently, thiol-targeted pharmacologic agents enable precise cysteine redox modulation: Controlled thiol oxidants such as diamide induce protective SSG, but risk off-target overoxidation; reversible covalent inhibitors, such as IBD-0063 selectively engage hyperreactive cysteines including PKM2 Cys358 for anticancer effects (94); natural electrophiles typified by sulforaphane activate NRF2 to upregulate endogenous GRX and glutathione systems, countering ferroptosis in redox-deficient pathologies (67). Notwithstanding promising preclinical outcomes, clinical translation confronts limitations in tissue selectivity, transient efficacy, and biomarker availability. Future research is required to prioritize mechanistic studies to elucidate the crosstalk between SSG and programmed cell death pathways, particularly its organelle-specific redox regulation in mitochondria, endoplasmic reticulum, and lysosomes. Translational efforts must focus on developing GRX mimetics and thiol-targeted pharmacological agents to restore SSG homeostasis in neurodegenerative disorders, cancer, and cardiovascular diseases. Additionally, integrative strategies combining traditional medicine-derived thiol modulators (e.g., tanshinone IIA) with advanced redox proteomics could unveil novel therapeutic targets and optimize precision medicine approaches for redox-related pathologies.

## Figures and Tables

**Table I:** Association between SSG and programmed cell death.

		The effects of SSG	
Type of cell death	The mediated pathways	Reaction-promoting mediators	Reaction-inhibiting mediators
Apoptosis	Endogenous pathways	BAX improves the opening of the mitochondrial permeability transition pore (MPTP) (35,36).	Bip impacts ATPase synthesis and the protein folding reaction (41) GSTP1, preventing protein misfolding (42).
	Exogenous pathways	FAS promotes the binding to FASL (37-39).	
	Endoplasmic reticulum pathways	Glutathione S-transferase pi 1 (GSTP1) inhibits the JNK signaling pathway (40).	
Autophagy	Macro autophagy	KEAPL1 inhibits the binding of NRF2(45),	ATG3 and ATG7 inhibit the lipidation of LC3(49).
	Micro autophagy Chaperone-mediated autophagy		SENP3 inhibits the formation of PtdIns3K (50).
Necroptosis	Extracellular signals Intracellular signals	MFN2 promotes the RIPK1- RIPK3-pMLKL complex formation (57,58).	Caspase-8 inhibits the phospho- rylation of MLKL (59).
Pyroptosis	Cascade inflammatory response	GSDMD promotes caspase-1 cleavage reaction (63).	NLRP3 undergoes redox mod-i fications (62).
Ferroptosis	Lipid peroxidation	GSH reduces GPX4 expression (64).	GRXs promote the biosynthesis of Fe-S clusters (66,67).
Disulfidptosis	Disulfide stress	Currently unknown	

The numbers in parentheses indicate reference citations. SSG, S-glutathionylation; GSDMD, gasdermin D; GRX, glutaredoxin; GSH, glutathione; GPX4, glutathione peroxidase 4.

**Table II:** Association between SSG and various SSG diseases.

Type of disease	Mechanisms of SSG on protein function	Influence pathways
Neurodegenerative diseases	Influence the activation or inactivation of key enzymes	The brain tissue p-SSG ratio was increased (70,71). GRX1 levels are downregulated in dopaminergic neurons (76).
	Affect the structural stability and degra- dation rate of target proteins	Disruption of F-actin protein nanostructures (72,75) (71,73).
		Reduction of GRX1 reverses mitochondrial oxidative damage (77).
Cardiovascular system diseases	Influence the activation or inactivation of key enzymes	Intramitochondrial NADPHase and GRX2 regulate mitochondrial ROS production (79).
	Affect the structural stability and degra dation rate of target proteins	Glutathionylation of SERCA proteins to produce excess ROS/RNS can cause Ml and l/R (83,84).
Respiratory system diseases	Influence the activation or inactivation of key enzymes	Overexpression of GRX1 increases lung epithelial cell survival (86).
	Affect the structural stability and degra- dation rate of target proteins	Reduced macrophage chemokine production via the NF-kB pathway (89).
Malignant tumor	Influence the activation or inactivation of key enzymes	SSG of the rate-limiting enzyme PMK2 to inhibit tumor growth (94).
	Alter protein conformation	Enhanced GSTP SSG is effective against MM resistance to Btz (97).
	Affect the structural stability and degra- dation rate of target proteins	Promoting TGM2 protein degradation improves the antitumor activity of drugs (95).

The numbers in parentheses indicate reference citations. SSG, S-glutathionylation; ROS, reactive oxygen species; RNS, reactive nitrogen species; GRX, glutaredoxin; GSTP, glutathione S-transferase Pi.

## Data Availability

Not applicable.
